# β2-microglobulin as a biomarker of pulmonary fibrosis development in COPD patients

**DOI:** 10.18632/aging.202266

**Published:** 2020-12-21

**Authors:** Zhenchao Wu, Mengdie Yan, Min Zhang, Nan Wu, Guoyuan Ma, Bingbing Wang, Youbo Fan, Xintong Du, Can Ding, Yi Liu

**Affiliations:** 1 Department of Pulmonary and Critical Care Medicine, Shandong Provincial Hospital, Cheeloo College of Medicine, Shandong University, Jinan 250021, Shandong, China; 2 Department of Thoracic Surgery, Shandong Provincial Hospital Affiliated to Shandong First Medical University, Jinan 250021, Shandong, China

**Keywords:** COPD, β2-microglobulin, pulmonary fibrosis, DLCO, diffusion

## Abstract

Expression of β2-microglobulin (β2M) is involved in fibrosis progression in kidney, liver, and heart. In this case-controlled retrospective study, we investigated the role of β2M in the development of pulmonary fibrosis in patients with chronic obstructive pulmonary disease (COPD). Analysis of 450 COPD patients revealed that patients with decreased pulmonary diffusing capacity (DLCO) had increased β2M serum levels. Compared to patients with lower β2M serum levels, patients with increased β2M levels exhibited increased alveolar wall/septal thickening and lung tissue β2M expression. In addition, patients with increased β2M levels had increased lung expression of TGF-β1, Smad4, and a-SMA. Animal experiments showed that increased β2M expression resulted in epithelial-mesenchymal transition (EMT), alveolar wall/septal thickening, and pulmonary fibrosis in a rat COPD model. Together, these results indicate that β2M serum levels may serve as a new indicator for assessment of pulmonary diffusion function and pulmonary fibrosis severity in clinical practice and may provide a potential target for treatment of pulmonary fibrosis in the future.

## INTRODUCTION

β2-Microglobulin (β2M) is the light chain of the class I major histocompatibility complex (MHC I) protein that plays an important role in innate and adaptive immunity, and is involved in the pathogenesis of respiratory diseases [1, 2]. Some studies have suggested that β2M may function as an inflammatory cytokine, aging-promoting factor, and fibrosis-related factor [3–8]. In addition, β2M is involved in the development of fibrosis in kidney, heart, and liver [9–12]. For example, under pressure overload, β2M promotes myocardial fibrosis and activation of myocardial fibroblasts [13]. Furthermore, β2M plasma levels are increased in patients with chronic hepatitis B cirrhosis [14]. These studies indicate that β2M may play a similar role in other organs, but its role in the development and progression of pulmonary fibrosis is not clear. Pulmonary fibrosis is a pathological change characterized by chronic nonspecific inflammation of pulmonary interstitial and large amounts of collagen deposition. In clinical practice, it is assessed by chest computed tomography (CT) and pulmonary diffusing capacity (DLCO) [15, 16]. Chronic obstructive pulmonary disease (COPD) is a complicated pulmonary and systemic inflammatory disease. Its pathogenesis involves pro-inflammatory cells and mediators that may lead to alveolar wall destruction and epithelial-mesenchymal transition (EMT), i.e. emphysema and fibrosis [17–20]. A recent study has shown that COPD patients with emphysema have increased β2M levels in plasma and lungs, suggesting a potential involvement of lung macrophages [21]. Significantly, patients with moderate and severe COPD often have a decreased diffusion capacity and pulmonary fibrosis that might be mediated by an inflammatory factor, such as β2M. In this study, we tested the hypothesis that β2M promotes lung fibrosis in COPD patients, and that it may serve as a novel biomarker of pulmonary fibrosis development in COPD patients.

## RESULTS

### COPD patients’ characteristics

This case-controlled, retrospective study included 450 COPD patients. Analysis of β2M levels and basic demographic information, systemic inflammatory levels, and pulmonary functions showed that β2M levels exhibited no correlation with gender, smoking index, BMI, RV/TLC, FEV1%pred, and PEF. However, the β2M levels showed a positive correlation with age, CRP levels, EOS%, FEV1/FVC, and MMEF, and a negative correlation with WBC, DLCO, and DLCO/V_A_ (Table 1).

**Table 1:** Spearman bivariate correlations analysis between β2M and basic demographic information, systemic inflammatory level, pulmonary function in COPD patients.

**Correlation**	**β2m**		
	**r**	**p-value**	**cases**
Gender	-0.083	0.077	450
Age	0.295	0.0001	450
Smoking index	0.043	0.365	450
BMI	-0.008	0.871	450
CRP	0.212	0.0002	296
WBC	-0.105	0.026	450
EOS%	0.175	0.0002	450
RV/TLC%	-0.056	0.244	441
FEV1%pred	0.087	0.065	449
FEV1/FVC%	0.146	0.002	443
PEF%	-0.009	0.853	449
MMEF%	0.111	0.02	441
DLCO%	-0.147	0.002	450
DLCO/VA%	-0.107	0.023	450
**Correlation after PSM**	**β2m**		
	**r**	**p-value**	**cases**
DLCO	-0.277	<0.001	186
DLCO/VA%	-0.128	0.081	186

Notes: P<0.05 was thought that the two indicators were significantly correlated, while r indicates correlation coefficient. Propensity score matching (PSM) were performed between lower β2m and higher β2m to exclude effect of unmatched baseline.

Abbreviations: BMI, body mass index; CRP, C reactive protein; WBC, white blood cell; EOS%, percentage of eosinophils in white blood cell; RV/TLC%, ratio of residual volume to total lung capacity; FEV1, forced expiratory volume in 1 second; FEV1%pred, percentage of forced expiratory volume in 1 second predicted value; FVC, forced vital capacity; FEV1/FVC%, percentage of FEV1/FVC; PEF%, percentage of peak expiratory flow; MMEF%, percentage of maximal mid-expiratory flow; DLCO%, percentage of diffusing capacity of carbon monoxide; DLCO/VA%, ratio of carbon monoxide diffusion capacity to alveolar ventilation; β2m,β2-Microglobulin; PSM, propensity score matching.

### β2M levels differ in COPD patients with better and worse DLCO values

The COPD patients were divided into four groups according to their DLCO values. The β2M levels gradually increased from DLCO I group to DLCO IV group (Table 2). Since there might be a β2M cut-off point, the COPD patients were divided into two groups – a “better DLCO group” (DLCO I and DLCO II) and a “worse DLCO group” (DLCO III and DLCO IV). Using Mann-Whitney U test, we found that the baseline characteristics were unmatched. The serum β2M values were 2.10±1.22 mg/L in the better DLCO group, and 2.24±0.89 mg/L in the worse DLCO group (Table 3). Using propensity score matching (PSM) to match the baseline, the β2M differences between the two groups were still significant (Table 3).

**Table 2:** Differences of clinical characteristics among four groups divided by DLCO in COPD patients.

	**DLCO I n=34**	**DLCO II n=82**	**DLCO III n=137**	**DLCO IV n=197**	**p-value**
Gender/male	67.60%	70.70%	82.50%	86.80%	0.003
Age, years	64.00 (59.75-70.25)	63.00 (58.00-70.00)	67.00 (60.00-76.00)	70.00 (63.00-76.00)	<0.001
Smoking index	0.00 (0.00-600.00)	155.00 (0.00-600.00)	600.00 (0.00-1000.00)	800.00 (200.00-1200.00)	<0.001
BMI, kg/m^2^	26.41 (22.80-30.95)	25.71 (23.39-30.49)	24.34 (21.62-27.34)	22.23 (19.53-25.08)	<0.001
CRP, mg/L	1.59 (0.56-5.57)	4.79 (1.69-27.59)	3.45 (1.43-11.76)	8.30 (2.60-27.03)	0.002
WBC, *10^9^/L	7.24 (5.41-8.91)	7.68 (5.93-10.67)	6.95 (5.66-8.91)	7.17 (5.65-8.85)	0.225
EOS%	1.55 (0.38-2.50)	0.95 (0.20-2.53)	1.90 (0.40-3.70)	1.60 (0.10-3.15)	0.194
β2m, mg/L	1.83 (1.48-2.11)	1.87 (1.56-2.48)	2.01 (1.74-2.44)	2.03 (1.73-2.54)	0.007

Notes: Data was presented as median (interquartile range, IQR) or proportion. P<0.05 indicated that the indicator was statistically different among groups.

Abbreviations: BMI, body mass index; CRP, C reactive protein; WBC, white blood cell; EOS%, percentage of eosinophil in white blood cell; β2m, β2-Microglobulin.

**Table 3:** Differences of clinical characteristics between better DLCO group (DLCO I and DLCO II) and worse DLCO group (DLCO III and DLCO IV) in COPD patients.

	**better DLCO n=116**	**lower DLCO n=334**	**p-value**
Gender/male	68.90%	85%	<0.001
Age, years	63.00 (58.25-70.00)	68.00 (62.00-76.00)	<0.001
Smoking index	100.00 (0.00-600.00)	637.50 (100.00-1012.50)	<0.001
BMI, kg/m^2^	26.08 (23.34-30.49)	22.99 (20.54-26.03)	<0.001
CRP, mg/L	3.72 (1.08-24.18)	5.17 (2.07-17.55)	0.248
WBC, *10^9^/L	7.35 (5.78-9.96)	6.99 (5.66-8.85)	0.119
EOS%	1.15 (0.2000-2.50)	1.65 (0.20-3.33)	0.104
β2m, mg/L	2.10±1.22	2.24±0.89	0.001
**After PSM**	**better DLCO n=93**	**lower DLCO n=93**	**p-value**
Male / Female	69/24	72/21	0.608
Age, years	64 (59-70)	64 (59-72)	0.879
Smoking index	200 (0-600)	400 (0-800)	0.274
BMI, kg/m^2^	26.09 (23.44-30.48)	22.84 (20.62-26.91)	<0.001
CRP, mg/L	3.38 (0.79-12.02)	5.08 (1.47-16.71)	0.189
β2m, mg/L	1.83 (1.51-2.31)	2.06 (1.79-2.45)	0.001
RV/TLC %	127.80 (114.75-144.75)	146.30 (121.15-162.80)	0.001
FEV1%pred	59.50 (47.35-71.00)	38.60 (30.20-46.35)	<0.001
FEV1/FVC%	62.15 (54.54-66.56)	51.09 (42.30-57.32)	<0.001
MMEF %	23.50 (17.00-33.40)	12.95 (10.25-18.28)	<0.001
DLCO %	70.90 (65.65-82.45)	39.00 (31.45-50.90)	<0.001
DLCO/VA %	85.80 (69.20-107.45)	48.60 (35.00-69.50)	<0.001
Emphysema	18.3%	43.0%	<0.001

Notes: Data was presented as median (interquartile range, IQR) or proportion or median±standard deviation. P<0.05 indicated that the indicator was statistically different between two groups. Propensity score matching (PSM) were performed between better DLCO and worse DLCO two groups to exclude effect of unmatched baseline.

Abbreviations: BMI, body mass index; CRP, C reactive protein; WBC, white blood cell; EOS%, percentage of eosinophil in white blood cell; β2m, β2-Microglobulin. PSM, propensity score matching; RV/TLC%, ratio of residual volume to total lung capacity; FEV1, forced expiratory volume in 1 second; FEV1%pred, percentage of forced expiratory volume in 1 second predicted value; FVC, forced vital capacity; FEV1/FVC%, percentage of FEV1/FVC; MMEF%, percentage of maximal mid-expiratory flow; DLCO%, percentage of diffusing capacity of carbon monoxide; DLCO/VA%, ratio of carbon monoxide diffusion capacity to alveolar ventilation.

### DLCO values differ in COPD patients with higher and lower β2M levels

In secondary analysis using the differences in β2M levels in the two DLCO groups, a cut-off point was set as 2.20 mg/L. Patients’ data were divided into a higher β2M group (serum β2M ≥2.20 mg/L) and a lower β2M group (serum β2M <2.20 mg/L). There were differences in age, CRP levels, and DLCO values between the two groups. COPD patients with emphysema represented less than 50% cases. Emphysema and RV/TLC % showed no differences between the two groups (Table 4).

**Table 4:** Differences of clinical characteristics between higher β2M group and lower β2M group in COPD patients.

	**lower β2m <2.20 n=125**	**higher β2m ≥2.20 n=61**	**p-value**
Male / Female	92/33	49/12	0.316
Age, years	62 (59-69)	66 (61.5-75)	0.001
Smoking index	400 (0-800)	300 (0-800)	0.963
BMI, kg/m^2^	25.1 (22.06-28.50)	24.77 (21.39-28.97)	0.873
CRP, mg/L	3.36 (0.74-8.32)	14.36 (3.17-92.32)	<0.001
RV/TLC %	134.45 (118.23-155.35)	134.60 (116.83-155.28)	0.992
FEV1%pred	46.50 (37.95-63.35)	49.70 (34.75-58.20)	0.614
FEV1/FVC%	55.48 (45.64-63.70)	56.06 (49.74-64.30)	0.632
MMEF %	16.70 (12.00-25.05)	18.25 (12.05-26.75)	0.719
DLCO %	62.50 (43.75-73.65)	49.30 (32.20-66.55)	0.003
DLCO/VA %	71.40 (56.45-91.85)	63.20 (40.85-95.15)	0.136
Emphysema	44.1%	40.7%	0.490

Notes: Data was presented as median (interquartile range, IQR) unless specified. P<0.05 indicated that the indicator was statistically different between two groups.

Abbreviations: β2m, β2-Microglobulin; BMI, body mass index; CRP, C reactive protein; RV/TLC%, ratio of residual volume to total lung capacity; FEV1, forced expiratory volume in 1 second; FEV1%pred, percentage of forced expiratory volume in 1 second predicted value; FVC, forced vital capacity; FEV1/FVC%, percentage of FEV1/FVC; MMEF%, percentage of maximal mid-expiratory flow; DLCO%, percentage of diffusing capacity of carbon monoxide; DLCO/VA%, ratio of carbon monoxide diffusion capacity to alveolar ventilation.

Referring to the results of DLCO and β2M subgroups, the indicators (age, BMI, CRP, and β2M) with p-values < 0.05 were selected into Binary logical regression model (Table 5). Higher BMI was better for COPD patients’ diffusion capacity (OR 0.841), while higher serum β2M was harmful to COPD patients’ diffusion capacity (OR 4.050). The model indicated that an increased serum β2M level was an independent risk factor for diffusion impairment and pulmonary fibrosis development in COPD patients.

**Table 5:** Binary logical regression for secondary analysis of indicators in COPD patients.

**DLCO**	**p-value**	**OR**	**OR 95% CI**
β2M	0.001	4.050	1.739-9.431
age	0.613	0.987	0.940-1.037
CRP	0.143	0.995	0.988-1.002
BMI	<0.001	0.841	0.765-0.924

Notes: P<0.05 indicated that the indicator was statistically different.

Abbreviations: DLCO, diffusing capacity of carbon monoxide; β2m, β2-Microglobulin; CRP, C reactive protein; BMI, body mass index; OR, odds ratio; 95% CI, 95% confidence interval.

### β2M serum and lung levels are associated with fibrosis in COPD patients

We measured β2M serum levels in 6 patients with COPD diagnosed by pulmonary function test, and analyzed their lung tissues collected during lung surgery. Patients with higher serum β2M levels had lower DLCO values compared to patients with low β2M levels. There was no difference in emphysema between the two groups; however, fibrosis was significantly different between the two groups, as reported by chest CT (Table 6).

**Table 6:** Differences of clinical characteristics between higher β2M group and lower β2M group in COPD patients which recruited from thoracic surgery department.

	**COPD patients with higher β2m n=3**	**COPD patients with lower β2m n=3**	**p-value**
Male, n	3	3	-
Age, years	62.33±8.31	58±4.98	0.189
Smoking index	440±435.25	800±715.54	0.746
BMI, kg/m^2^	27.1±2.33	27.34±3.05	0.746
β2m, mg/L	2.45±0.28	1.56±0.36	0.004
DLCO %	65.8±13.19	94.57±24.02	0.023
DLCO/VA %	91.27±10.65	110.97±26.96	0.106
Emphysema, n	0	1	0.138
Fibrosis, n	3	0	0.001

Notes: Data was presented as mean ± standard deviation. P<0.05 indicated that the indicator was statistically different between two groups.

Abbreviations: BMI, body mass index; β2m, β2-Microglobulin; DLCO%, percentage of diffusing capacity of carbon monoxide; DLCO/VA%, ratio of carbon monoxide diffusion capacity to alveolar ventilation.

**Figure 1:**
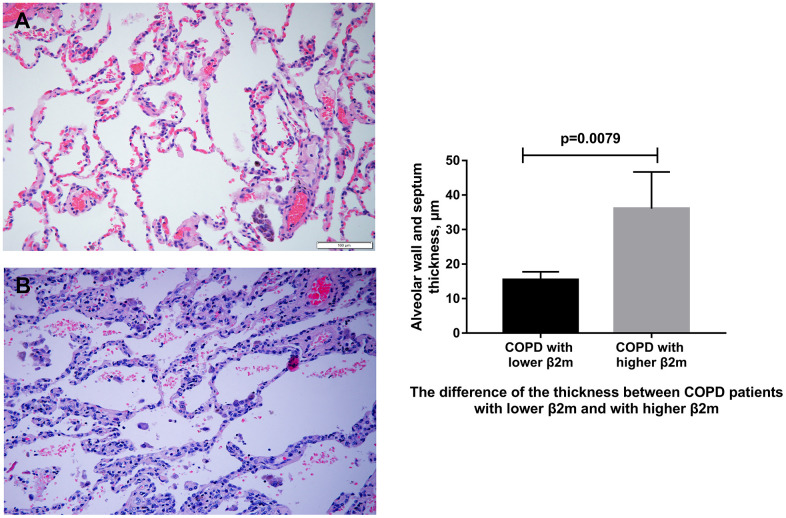
**HE staining of lung tissue from COPD patients.** (**A**) Representative image of HE staining of lung tissue from COPD patients with lower serum β2M. (**B**) Representative image of HE staining of lung tissue from COPD patients with higher serum β2M. The right panel shows difference of alveolar wall/septum thickness in COPD patients with lower serum β2M and with higher serum β2M. P<0.05 in COPD patients with higher serum β2M versus those with lower serum β2M. Bars represent Means.

**Figure 2:**
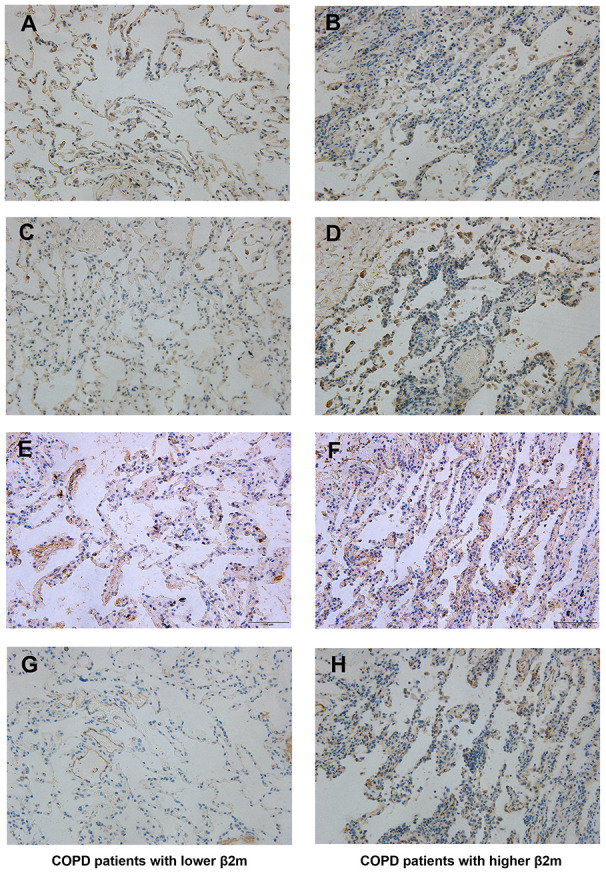
**Immunohistochemical staining of lung tissue from COPD patients.** Lung tissue of COPD patients with lower serum β2M including (**A**, **C**, **E**, **G**); lung tissue of COPD patients with higher serum β2M including (**B**, **D**, **F**, **H**). Indicators and positive cell rate: (**A**, **B**) Representative image of β2M immunohistochemical staining of lung tissue from COPD patients with lower serum β2M (17.17 ± 1.64%) and with higher serum β2M (28.95 ± 1.26%) respectively. (**C**, **D**) Representative image of TGF-β1 immunohistochemical staining of lung tissue from COPD patients with lower serum β2M (16.48 ± 0.63%) and with higher serum β2M (32.46 ± 0.69%) respectively. (**E**, **F**) Representative image of Smad4 immunohistochemical staining of lung tissue from COPD patients with lower serum β2M (34.95 ± 0.71%) and with higher serum β2M (43.38 ± 0.90%) respectively. (**G**, **H**) Representative image of a-SMA immunohistochemical staining of lung tissue from COPD patients with lower serum β2M (3.854 ± 0.43%) and with higher serum β2M (26.66 ± 0.89%) respectively. P<0.05 in COPD patients with higher serum β2M versus those with lower serum β2M.

Using hematoxylin-eosin (HE) staining, we found that the COPD patients with higher serum β2M levels had thicker alveolar wall/septum (Figure 1). Immunohistochemical (IHC) staining showed that the patients with increased serum β2M levels had an increased β2M expression in lung tissues, especially in the alveolar wall/septum. In addition, they had increased expression of TGF-β1, Smad4, and a-SMA compared to patients with low serum β2M levels (P<0.05; Figure 2).

### β2M lung expression correlates with EMT and fibrosis progression in COPD rats

**Figure 3:**
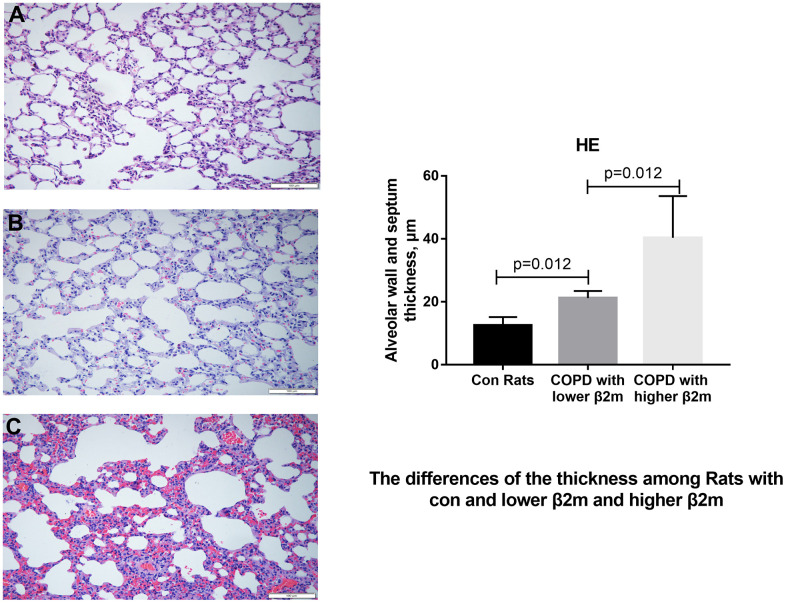
**HE staining of lung tissue from rats.** (**A**–**C**) Representative image of HE staining of lung tissue from control rats, COPD rats with lower β2M and COPD rats with higher β2M, respectively. The right panel shows quantification of alveolar wall and septum thickness in control rats, COPD rats with lower β2M and COPD rats with higher β2M. P<0.05 in COPD rats with lower β2M versus control and in COPD rats with higher β2M versus COPD rats with lower β2M. Bars represent Means.

**Figure 4:**
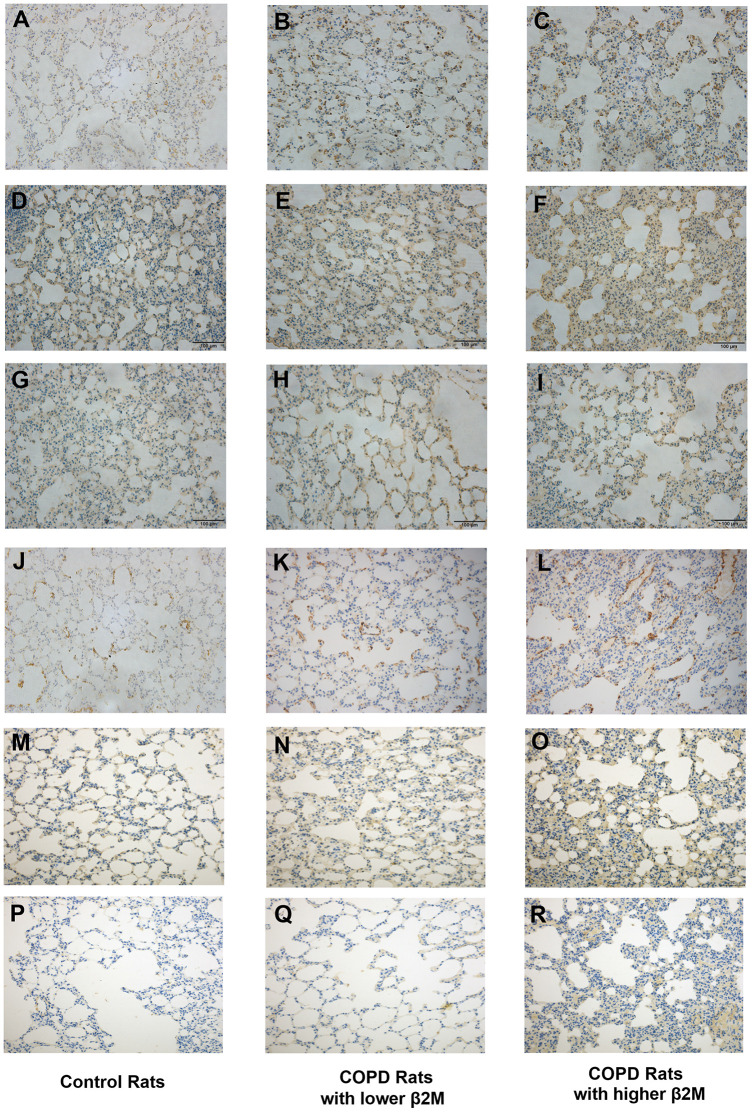
**Immunohistochemical staining of lung tissue from rats.** Lung tissue of control rats including (**A**, **D**, **G**, **J**, **M**, **P**); lung tissue of COPD rats with lower β2M including (**B**, **E**, **H**, **K**, **N**, **Q**); lung tissue of COPD rats with higher β2M including (**C**, **F**, **I**, **L**, **O**, **R**). Indicators and positive cell rate: (**A**–**C**) Representative image of β2M immunohistochemical staining of lung tissue from control rats (7.29 ± 1.65%), COPD rats with lower β2M (14.39 ± 2.17%) and COPD rats with higher β2M (21.21 ± 2.56%) respectively. (**D**–**F**) Representative image of TGF-β1 immunohistochemical staining of lung tissue from control rats (30.12 ± 3.24%), COPD rats with lower β2M (33.22 ± 2.87%) and COPD rats with higher β2M (37.30 ± 4.99%) respectively. (**G**–**I**) Representative image of Smad4 immunohistochemical staining of lung tissue from control rats (20.67 ± 2.25%), COPD rats with lower β2M (27.04 ± 2.99%) and COPD rats with higher β2M (29.51 ± 3.14%) respectively. (**J**–**L**) Representative image of a-SMA immunohistochemical staining of lung tissue from control rats (5.82 ± 0.57%), COPD rats with lower β2M (7.99 ± 1.35%) and COPD rats with higher β2M (9.96 ± 3.10%) respectively. (**M**–**O**) Representative image of col1 immunohistochemical staining of lung tissue from control rats (8.43 ± 2.58%), COPD rats with lower β2M (13.19 ± 7.05%) and COPD rats with higher β2M (19.67 ± 7.46%) respectively. (**P**–**R**) Representative image of col3 immunohistochemical staining of lung tissue from control rats (12.53 ± 8.96%), COPD rats with lower β2M (12.57 ± 7.06%) and COPD rats with higher β2M (22.04 ± 10.14%) respectively. The pictures show the same differential tendency (P<0.05).

**Figure 5:**
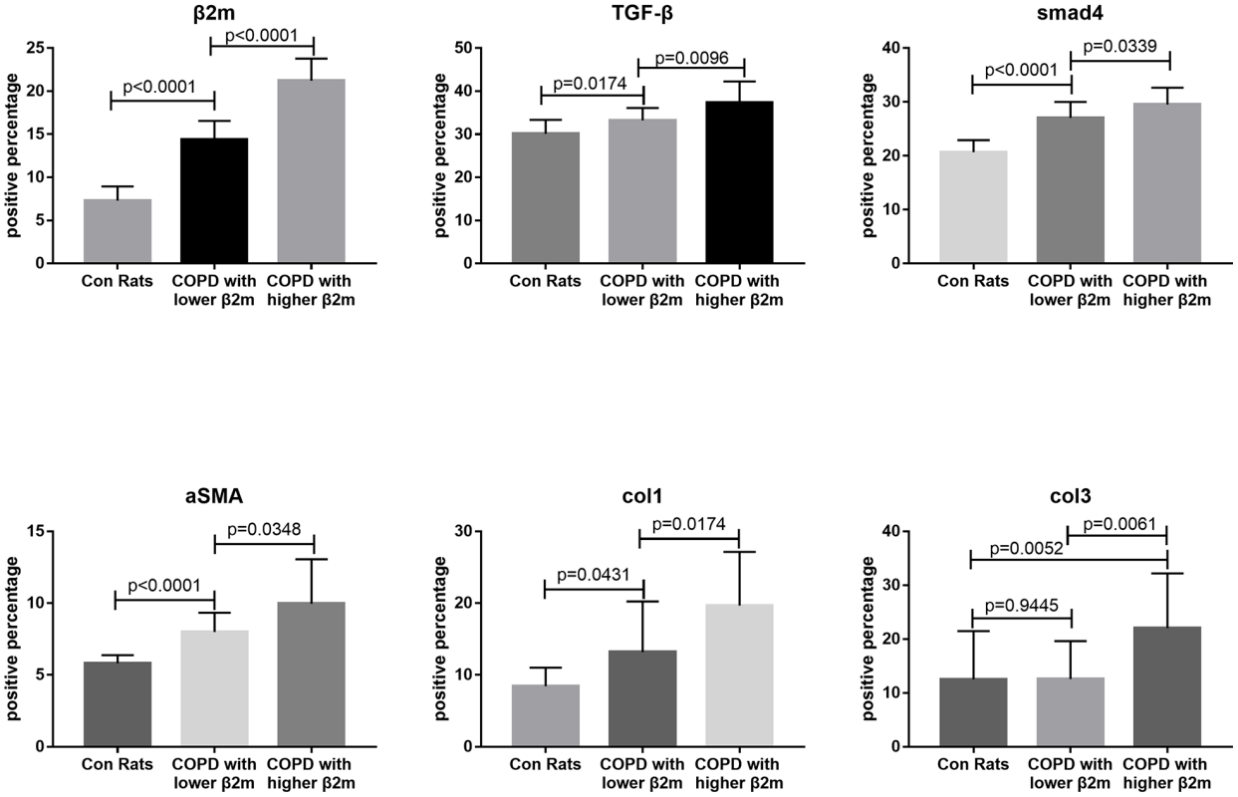
**Immunohistochemical staining quantitative traits of lung tissue from rats.** This figure means quantified Figure 4. Every Each individual bar chart is factor immunohistochemical staining quantitative traits of lung tissue from rats. Factors including: β2M, TGF-β1, Smad4, a-SMA, col1, col3. P<0.05 represents difference is significant.

**Figure 6:**
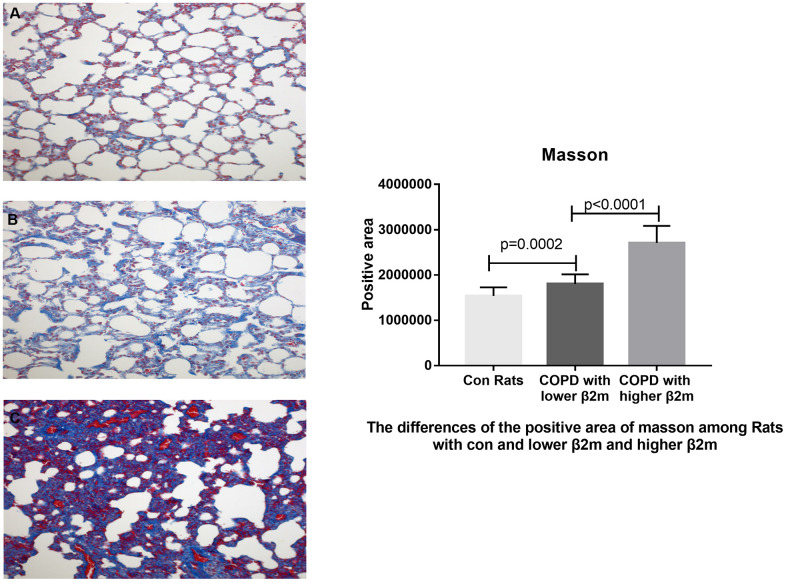
**Masson's trichrome staining of lung tissue from rats.** (**A**–**C**) Representative image of Masson's trichrome staining of lung tissue from control rats, COPD rats with lower β2M and COPD rats with higher β2M, respectively. The right panel shows Masson staining positive area in control rats, COPD rats with lower β2M and COPD rats with higher β2M. P<0.05 in COPD rats with lower β2M versus control and in COPD rats with higher β2M versus COPD rats with lower β2M. Bars represent Means.

To rule out the effect of other diseases, such as lung cancer, aging, or obesity, we used a COPD rat model to validate our data indicating that increased β2M levels induce alveolar epithelial-mesenchymal transition (EMT) and pulmonary fibrosis in COPD. HE staining (Figure 3) showed that control rats (n=6) had normal alveolar construction (X_A_=12.52μm), while COPD rats had moderately thick alveolar wall/septum (XB=21.22μm, n=7) and COPD rats had thickest alveolar wall/septum (XC=40.38μm, n=6). IHC staining demonstrated that the lung tissues of 6 COPD rats with the thickest alveolar wall/septum exhibited increased β2M expression compared to 7 COPD rats with moderately thick alveolar wall/septum and 6 rats with normal alveolar construction. In addition, the lung expression of TGF-β1, Smad4, a-SMA, col1, and col3 showed the same tendency as the β2M expression among the three rat groups, P<0.05 (Figures 4, 5). Masson staining showed that the 6 COPD rats with the thickest alveolar wall had a massively increased expression of collagen fibers in their lung tissues (Figure 6).

## DISCUSSION

Due to β2M structural characteristics, this amyloid protein can be distributed by blood to all parts of the body, deposit in various tissues and organs, and cause varying degrees of destruction [22]. β2M has been used as a biomarker of fibrosis progression in several organs, including kidney, heart, and liver [6, 7, 9, 12]. However, its role in the lungs has been insufficiently studied. COPD is a complicated pulmonary and systematic disease whose mechanisms involve inflammation, autophagy, aging, and EMT/fibrosis [23]. Moderate and severe COPD patients often have a lung diffusion impairment that is characterized by alveolar epithelial cells EMT, alveolar wall/septal thickening, and alveoli capillary membrane damage detected by pulmonary function test (DLCO and/or DLCO/VA) [24–30]. A recent study has indicated that β2M expression is increased in alveolar epithelial cells, suggesting that β2M might be involved in COPD progression [21]. In this retrospective study, we tested the hypothesis that increased β2M serum and lung levels lead to diffusion dysfunction and pulmonary fibrosis development (DLCO decreasing, alveolar-related EMT, lung tissue fibrosis) in COPD patients.

To our knowledge, our study is the first to demonstrate that COPD patients with decreased DLCO values have increased serum β2M levels. Analysis of clinical data indicated that increased serum β2M is a harmful factor for DLCO in COPD patients, while BMI is a protective one. The observed positive effect of BMI on DLCO is in line with our previous study [31]. In addition, our results showed that increased serum and lung levels of β2M correlated with increased alveolar wall/septal thickening (fibrosis changes) in COPD patients. The TGF-β/Smad pathway is a classical EMT mechanism, and a-SMA, col1, and col3 levels are reliable indicators of EMT/fibrosis progression [18, 32–35]. We investigated the mechanisms that contribute to EMT and fibrosis progression in COPD, and hypothesized that high β2M expression could stimulate TGF-β1 expression, resulting in increased Smad4 and a-SMA levels and collagen expression. Analysis of clinical samples demonstrated that COPD patients with increased serum and lung β2M levels had increased expression of TGF-β1, Smad4, and a-SMA, and increased alveolar wall/septal thickness, resulting in lower DLCO values and fibrosis changes. Animal experiments showed alveolar wall/septal thickening, and increased levels of β2M, TGF-β1, Smad4, a-SMA, col1, and col3 in a rat COPD model. In addition, COPD rats exhibited a significant Masson staining in lung tissues, suggesting leukocyte-mediated pulmonary inflammatory response and then pulmonary fibrosis development.

Future studies should investigate the specific stimuli and mechanisms that induce the increased β2M expression in the lungs and in serum, and elucidate the potential involvement of lung inflammatory cells. It will be important to determine the mechanisms regulating the β2M expression, its relationship with pulmonary fibrosis, as well as the potential of β2M protein structure to contribute to fibrosis.

In conclusion, our study demonstrates the novel relationship between β2M levels, and EMT and lung fibrosis in COPD patients. Our data show that the increased β2M expression is mediated by the TGF-β1/Smad4/a-SMA pathway, resulting in alveolar epithelial cell EMT, alveolar wall/septal thickening, pulmonary fibrosis, and decreased DLCO values. These findings indicate that serum β2M levels may serve as a new indicator to assess pulmonary diffusion function and pulmonary fibrosis severity in clinical practice, and suggest that β2M may serve as a novel potential intervention target for the treatment of pulmonary fibrosis.

## MATERIALS AND METHODS

### Study population

450 COPD patients were included in this case-control retrospective study. COPD was diagnosed by pulmonary function test according to the standards of Global Initiative for Chronic Obstructive Lung Disease (GOLD). Patients with chronic kidney disease (CKD) or other uncontrolled serious systemic disease were excluded. Patients’ data (containing basic demographic information, history of smoking, laboratory blood tests, and pulmonary function tests) were collected from Hospital electronic records between January 1, 2013 and December 31, 2017.

In addition, lung tissue specimens were obtained from 6 COPD patients (3 with higher serum β2M values and 3 with lower serum β2M), who were diagnosed with solitary lung nodule and needed resection. Then specimens were fixed by 10% formalin and embedded with paraffin for hematoxylin-eosin (HE) and immunohistochemical (IHC) staining. All procedures adhered to the Helsinki Declaration. This study was approved by Shandong Provincial Hospital Medical Ethics Committee (Ethical Review of Medical Research on Human Being No. 2016-23; LCYJ: NO. 2019-019).

### Rat COPD model

Adult male Wistar rats weighing above 200 g were used for all experiments. Rats were fed in SPF animal facility (temperature 22±2° C, humidity 55±5%), with normal day and night cycle, and free access to common diet and water. Rats were randomly divided into 2 groups: control (n=6) and COPD/CSE (n=13). All experimental procedures followed the Guidelines of the Institutional Animal Care and Use Committee, Chinese Academy of Sciences.

Rat model of COPD was exposed to cigarette smoke (10/time, Huangshan Brand, China Tobacco Anhui Industrial Co., Ltd.) for 4 times each day in a special device, with more than 3-hour breaks in between. The exposure was performed for 5 days per week, and lasted for 12 weeks before final measurements of lung function. The non-COPD groups were put in the same case with free access to fresh air. After exposure, all rats were fed in the room and had access to fresh air. After lung function was measured and COPD model was established, rats were sacrificed and the lung tissues were obtained. The study was approved by Shandong Provincial Hospital Animal Experiment Ethics Committee (Ethical Review of Animal Experiment No. 2019-001).

### Study design

We hypothesized that serum and tissue β2M values may serve as a biomarker of pulmonary fibrosis (determined by lung diffusion function - DLCO; chest CT; lung tissue HE staining - thickness of alveolar wall and thickness of alveolar septum; fibrosis indicators immunohistochemical staining of lung tissue; and Masson staining) in COPD. 450 COPD patients were divided into four groups according to their DLCO values (Normal group DLCO I≥80%, Mild Impairment group 80>DLCO II≥60%, Moderate Impairment group 60%>DLCO III≥40%, Severe Impairment group DLCO IV<40%). Then we divided these COPD patients into two groups: a better diffusing capacity group (combined Normal group and Mild Impairment group, DLCO≥60%) and a worse diffusing capacity group (combined Moderate Impairment group and Severe Impairment group DLCO<60%), and analyzed the differences in serum β2M values between these two groups. Secondary analysis was then performed to find a cut-off point of serum β2M to verify the difference in DLCO of COPD patients.

In addition, lung tissues and serum samples from 6 COPD patients (3 with high serum β2M and 3 with low serum β2M) and 19 rats (control n=6, COPD n=13) were analyzed by HE staining for changes in alveolar walls and alveolar septum, IHC staining of β2M and fibrosis indicators (TGF-β1, Smad4, α-SMA, col1, col3), and Masson staining for observing collagen fiber content in lung tissues.

### Histology and morphometric analyses

The lung tissues were fixed in 4% paraformaldehyde, embedded in paraffin, and cut into 4.5 μm-thick sections. The sections were stained with hematoxylin and eosin (HE). The measurement of distal airspace wall thickness was analyzed by using the Image Pro-Plus. In brief, the slides were observed at 200× magnification. The measurement was performed by drawing lines horizontally across the field of view. Bronchioles were intentionally ignored in the field. The thickness of each septum crossing the given horizontal line was measured perpendicular to its course at that crossing point. Three separate horizontal lines were drawn and analyzed for each field, and the average septal thickness was calculated for each grid. There were three lung specimens per group for analyses of distal airspace wall thickness.

### IHC staining

The 4.5 μm-thick sections obtained from paraffin-embedded lung tissues were deparaffinized, and antigen retrieval was performed at a temperature over 95° C. Nonspecific reactions were blocked with goat serum for 15 minutes at 37° C, and the sections were incubated with primary antibodies against β2-Microglobulin (Proteintech 13511-1-AP, Rosemont, IL, USA, 1:50), TGF-β1 (Proteintech 21898-1-AP, Chicago, Il, USA, 1:100), Smad4 (Proteintech 10231-1-AP, Chicago, Il, USA, 1:50), a-SMA (Proteintech 14395-1-AP, Rosemont, IL, USA, 1:3000), col1 (YM3767, Immonoway, Beijing, China, 1:50), and col3 (Proteintech 22734-1-AP, Sanying, Wuhan, China, 1:500) for 1 hour at 37° C. The sections were incubated with a secondary antibody kit (SZGB-BIO, PV9003, Beijing, China) for 20 minutes at 37° C. The samples were viewed under a confocal FV 1000 SPD laser scanning microscope (Olympus, Japan), and labeling index analyses were performed with ImageJ.

### Masson's trichrome staining

The sections were treated with Masson trichrome (MT) staining kit (Servicebio, G1006, Wuhan, China). Visual fields were randomly selected for each section, using the same conditions for light setting and contrast. Expression of collagen fibers in pulmonary alveoli was quantified by measuring the positive staining area using ImageJ.

### Statistical analysis

All data were collected from Hospital electronic records and recorded by EpiData CRF (Version 3.1). Statistical analysis was performed by SPSS Version 23.0 (SPSS Inc.; Chicago, Illinois) and figures were made by GraphPad Prism7 (GraphPad, San Diego, CA) and ImageJ (National Institutes of Health, USA). Correlation between β2M and DLCO, DLCO/V_A_ was analyzed by Spearman bivariate analysis. In the primary analysis, parametric data were presented as mean ± standard deviation or mean (range). Non-parametric data were presented as median (interquartile range, IQR). Kruskal-Wallis test was used to compare differences among multi-groups and Mann-Whitney U test was used to compare data between pairs. Propensity score matching (PSM) test was performed between DLCO better and DLCO worse groups to exclude the effect of unmatched baseline. Then logistic regression was used to analyze multivariable parameters. In the secondary analysis, analyzing the cut-off point to divide COPD patients into high β2M groups and normal β2M groups, the differences between β2M groups were analyzed by Mann-Whitney U test. P<0.05 was considered significant for all statistical analyses.

### Study approval

This study adhered to the Helsinki Declaration protocol, and was approved by Shandong Provincial Hospital Medical Ethics Committee (Ethical Review of Medical Research on Human Being No. 2016-23; LCYJ: NO. 2019-019). Animal experiments were approved by Shandong Provincial Hospital Animal Experiment Ethics Committee (Ethical Review of Animal Experiment No. 2019-001).
